# Photoperiodic regulation of the sucrose transporter StSUT4 affects the expression of circadian-regulated genes and ethylene production

**DOI:** 10.3389/fpls.2013.00026

**Published:** 2013-02-20

**Authors:** Izabela Chincinska, Konstanze Gier, Undine Krügel, Johannes Liesche, Hongxia He, Bernhard Grimm, Frans J. M. Harren, Simona M. Cristescu, Christina Kühn

**Affiliations:** ^1^Department of Plant Physiology, Institute of Biology, Humboldt University of BerlinBerlin, Germany; ^2^Department of Molecular and Laser Physics, Radboud University NijmegenNijmegen, Netherlands

**Keywords:** flowering, shade avoidance syndrome, sucrose transport, ethylene

## Abstract

Several recent publications reported different subcellular localization of the sucrose transporters belonging to the SUT4 subfamily. The physiological function of the SUT4 sucrose transporters requires clarification, because down-regulation of the members of the SUT4 clade had different effects in rice, poplar, and potato. Here, we provide new data for the localization and function of the Solanaceous StSUT4 protein, further elucidating involvement in the onset of flowering, tuberization and in the shade avoidance syndrome of potato plants. Induction of an early flowering and a tuberization in the SUT4-inhibited potato plants correlates with increased sucrose export from leaves and increased sucrose and starch accumulation in terminal sink organs, such as developing tubers. SUT4 affects expression of the enzymes involved in gibberellin and ethylene biosynthesis, as well as the rate of ethylene biosynthesis in potato. In the SUT4-inhibited plants, the ethylene production no longer follows a diurnal rhythm. Thus it was concluded that StSUT4 controls circadian gene expression, potentially by regulating sucrose export from leaves. Furthermore, SUT4 expression affects clock-regulated genes such as *StFT, StSOC1*, and *StCO*, which might be also involved in a photoperiod-dependent tuberization. A model is proposed in which StSUT4 controls a phloem-mobile signaling molecule generated in leaves, which together with enhanced sucrose export affects developmental switches in apical meristems. SUT4 seems to link photoreceptor-perceived information about the light quality and day length with phytohormone biosynthesis and the expression of circadian-regulated genes.

## Introduction

The physiological function of the members of the SUT4 family of sucrose transporters still remains unclear. Mutants or transgenic plants with reduced expression of the SUT4 genes do not show consistent phenotype in different plant species. In potato, inhibition of the StSUT4 expression by RNA interference is accompanied by early flowering and tuberization, as well as decreased sensitivity toward a far-red light enriched irradiation which might be due to increased sucrose export from transformant leaves at the end of the light period (Chincinska et al., 2008). In contrast, leaves of the *ossut2* mutant plants export less sucrose when compared to the wild type. As a consequence, sucrose, glucose, and fructose accumulate, resulting in growth retardation and reduced development of roots and grains (Atkins et al., 2011). Consistently with the observations in rice, the SUT4-homologue from poplar, PtaSUT4, also affects sucrose export from source leaves. The decreased translocation of sucrose to the sink organs in PtaSUT4-repressed poplar plants results in an increased leaf-to-stem biomass ratio (Payyavula et al., 2011). Thus, StSUT4 from potato seems to inhibit sucrose efflux from leaves, whereas OsSUT2 from rice and PtaSUT4 appear to promote sucrose export from source leaves under normal conditions. It should be noted that rice and poplar employ a different strategy for phloem loading when compared to potato (Eom et al., 2012). In rice, a passive mode of phloem loading via sucrose diffusion through plasmodesmata was postulated, with involvement of the vacuolar OsSUT2 as a valve regulating sucrose flux into the phloem (Eom et al., 2012).

The SUT4 clade is heterogeneous and includes also sucrose facilitators described in *Pisum sativum* (Zhou et al., 2007), as well as sucrose proton co-transporters described in poplar and tomato (Weise et al., 2000; Reinders et al., 2008). Despite these differences, sequence homologies revealed phylogenetic similarities between the different functional classes.

It is therefore questionable, whether a general conclusion about localization and function of members of the SUT4 family can be drawn for the different plant species.

Functional diversity is also proposed after comparison of promoter elements of homologous SUT4 genes. For example, whereas OsSUT2 promoters contain hormone-related elements (Washio, 2003), Arabidopsis AtSUC4 has mainly elements related to the stress-response (Ibraheem et al., 2010). The *cis*-regulatory elements of the promoter regions of different members of the SUT4 subfamily differ significantly between rice, Arabidopsis, and potato (Ibraheem et al., 2010). This leads to different expression patterns of the SUT4 homologues in different plant species. These observations, together with the fact that inhibiting the SUT4 activity in different plant species leads to either no phenotype (Arabidopsis) or to opposite effects on sucrose export (rice, poplar, and potato), are strong arguments against functional similarities between different members of the SUT4 subfamily.

In the current paper, we aim to provide new insight into the localization and function of SUT4 function in potato.

As mentioned above, plants with reduced *StSUT4* transcript levels exhibit an early flowering and tuberization phenotype and do not show the shade avoidance syndrome in response to increased red:far-red light ratio (Chincinska et al., 2008). *StSUT4* mRNA accumulates in response to shading and this increase in transcript accumulation is not due to the enhanced transcriptional activity but rather due to increased transcript stability, as shown by treatment with the transcriptional inhibitor actinomycin D (Liesche et al., 2011). This increased transcript stability seems to be under the control of phytochrome B, because *phyB* antisense potato plants exhibiting constitutive shade avoidance symptoms do not show differences in *StSUT4* transcript accumulation under white light or far-red light enriched conditions (Liesche et al., 2011).

A further observation made in *StSUT4* mutants were altered transcript levels of enzymes involved in GA and ethylene biosynthesis. It was concluded that flowering and tuberization in potato share common pathways (Rodriguez-Falcon et al., 2006; Kühn, 2011). Indeed, flowering-related genes, e.g., *StFT* or *StCO*, are also involved in the regulation of tuberization in potato plants (Navarro et al., 2011; Gonzalez-Schain et al., 2012).

The inhibitory effect of ethylene on tuberization was described earlier (Mingo-Castel et al., 1974). Recent publications revealed a key role of the ethylene-dependent pathway also in the control of flowering and shade avoidance (Samach et al., 2000; Wuriyanghan et al., 2009). Ethylene and gibberellic acid biosynthesis pathways are suspected to trigger the symptoms of the shade avoidance syndrome in tobacco plants (Pierik et al., 2004; Stamm and Kumar, 2010). Furthermore, a recent publication revealed the involvement of ethylene in the delay of flowering in Arabidopsis via a DELLA-dependent pathway (Achard et al., 2007). Therefore, the main goal of the present studies was to elucidate the role of StSUT4 in the process of flower and tuber induction including quantification of the circadian-regulated gene transcripts and determination of the ethylene synthesizing capacity.

## Results

### Subcellular localisation of solanaceae SUT4 proteins

**Figure 1:**
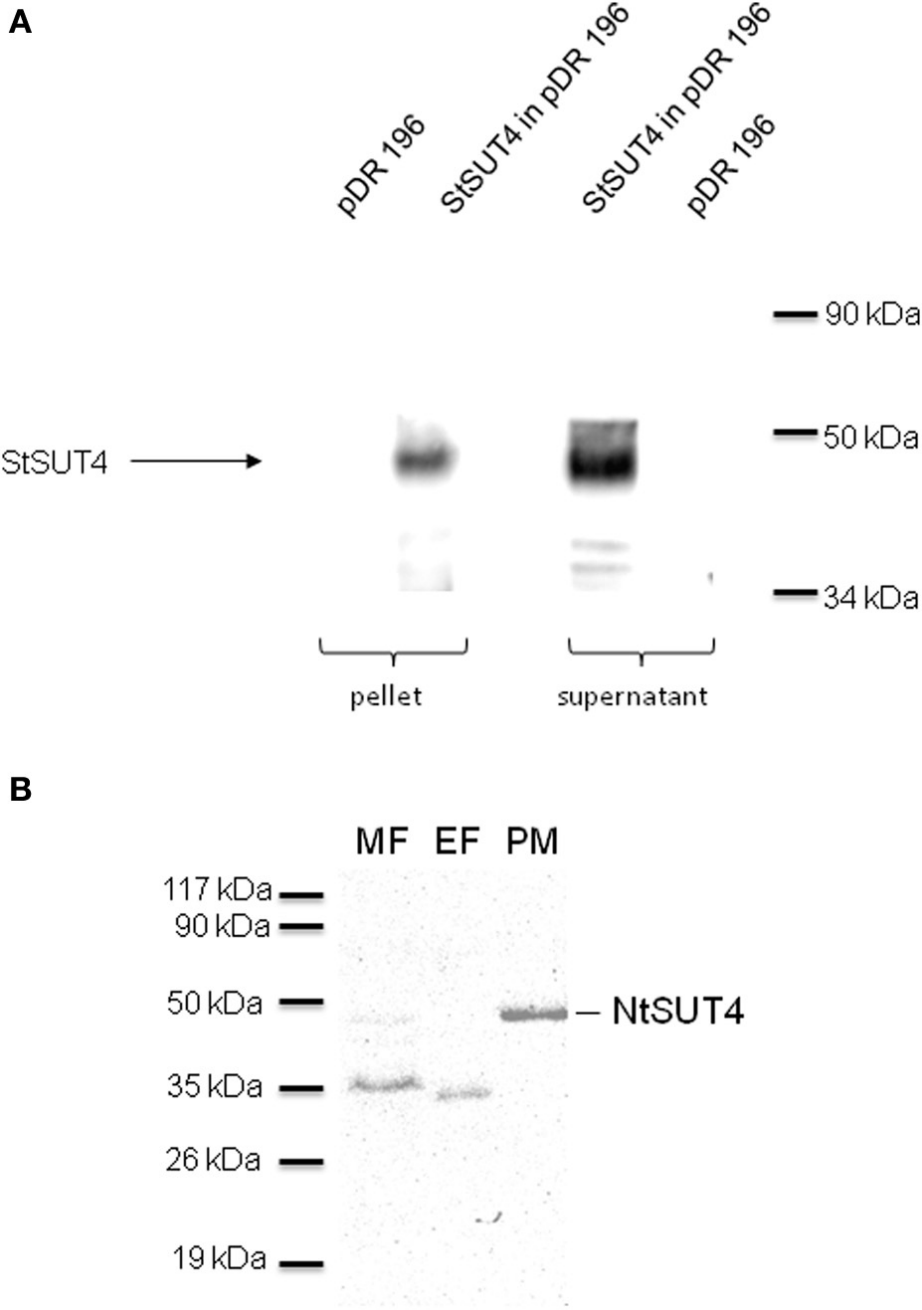
**(A)** Western Blot analysis of yeast strain SUSY7 expressing StSUT4. The specificity of the StSUT4-specific polyclonal antibody generated in rabbits was tested using microsomal fractions from yeast cells expressing StSUT4 in pDR196 or the empty vector control (pDR196). The corresponding low spin pellet as well as the supernatant was loaded with Laemmli sample buffer and separated by SDS-PAGE. A band of the expected size of 46 kDa is detectable only in yeast extracts expressing StSUT4. **(B)** Western blot analysis of NtSUT4 in different membrane fractions from tobacco source leaf material. The microsomal fraction (MF) was separated by two-phase partitioning into the endomembranes fraction (EF) and the plasma membrane fraction (PM) by two-phase partitioning as described earlier (Chincinska et al., 2008). 20 μg of protein were loaded per lane on a 12.5% SDS gel. A band with the expected size of 46 kDa was mainly detectable in the plasma membrane fraction.

SUT4 members have been reported to be targeted either to the plasma membrane or to the vacuole (Table 1). Only in one proteomic report AtSUT4 has been shown to reside in the chloroplast envelope (Rolland et al., 2003). StSUT4 localization in potato was determined in the plasma membrane of sieve elements using specific affinity-purified peptide antibodies (Weise et al., 2000). The specificity of the antibody was validated in plants (Chincinska et al., 2008) and in yeast cells expressing StSUT4 in the yeast expression vector pDR196 (Figure 1A) by western blot analysis. Fractionation of the microsomal fraction of potato source leaf material into the endomembrane fraction and the plasma membrane fraction revealed two distinct bands of different sizes: one band corresponding to the full length protein of approx. 46 kDa in the plasma membrane fraction and a second band corresponding potentially to a truncated version of the StSUT4 protein in the endomembranes fraction (Chincinska et al., 2008).

**Table 1:** **Functional characterization of members of the SUT4 subfamily of sucrose transporters reveals a different localization than GFP fusion**.

**Gene**	**Organism**	**Localisation**	**Based on**	**References**
AtSUT4	Arabidopsis	Plasma membrane	Functional characterization in yeast	Weise et al., 2000
AtSUT4 (SUC4)	Arabidopsis	Vacuole	Vacuole proteomics	Endler et al., 2006
AtSUT4	Arabidopsis	Chloroplast envelope	Chloroplast proteomics	Rolland et al., 2003
AtSUT4	Arabidopsis	Plasma membrane	Interaction with PM transporters	Schulze et al., 2003
HvSUT2	Barley	Plasma membrane	Functional characterization in yeast	Weschke et al., 2000
HvSUT2	Barley	Vacuole	GFP fusion, tonoplast proteomics	Endler et al., 2006
LjSUT4	Lotus	Plasma membrane	Functional characterization in Xenopus	Reinders et al., 2008
LjSUT4	Lotus	Vacuole	GFP fusion	Reinders et al., 2008
NtSUT4	Tobacco	Plasma membrane	Functional characterization in yeast	Okubo-Kurihara et al., 2011
NtSUT4	Tobacco	Vacuole	GFP fusion	Okubo-Kurihara et al., 2011
NtSUT4	Tobacco	Plasma membrane	Plant Western Blot	This work
PtaSUT4	Poplar	Plasma membrane	Plasma membrane proteomics	Nilsson et al., 2010
PtaSUT4	Poplar	Vacuole	GFP fusion	Payyavula et al., 2011
OsSUT2	Rice	Plasma membrane	Functional characterization in yeast	Eom et al., 2011
OsSUT2	Rice	Vacuole	GFP fusion	Eom et al., 2011
SlSUT4	Tomato	Plasma membrane	Immunolocalisation, GFP fusion	
SlSUT4	Tomato	ER, Vacuole	GFP fusion	Schneider et al., 2012
StSUT4	Potato	Plasma membrane	Functional characterization in yeast	Weise et al., 2000
StSUT4	Potato	Plasma membrane	Plant Western Blot, GFP fusion	Chincinska et al., 2008
StSUT4	Potato	ER	Split YFP	Krügel et al., 2012
StSUT4	Potato	PM, Vacuole	GFP fusion	This work
MdSUT1	Apple	Plasma membrane	Functional in yeast, GFP fusion, interaction with ER protein	Fan et al., 2009

All members of the SUT4 clade are targeted to at least two different compartments.

Recently, the subcellular localization of the homologous protein from *Nicotiana tabacum*, NtSUT4, was determined to be vacuolar, as shown by co-localization of the endocytosis marker FM4-64 in BY2 cells (Okubo-Kurihara et al., 2011). Nevertheless, NtSUT4 is able to complement a sucrose-uptake deficient yeast mutant strain which requires at least a small fraction of NtSUT4 to be localized at the plasma membrane (Okubo-Kurihara et al., 2011). Since the StSUT4-specific antibody is able to cross-react with NtSUT4, we performed subcellular fractionation of the microsomal membrane fraction of tobacco source leaves by two-phase partitioning, in order to separate the plasma membrane from the endomembranes fraction (Figure 1B). As previously shown for StSUT4 in potato, the full length NtSUT4 protein is only detectable in plasma membranes and a very faint band is visible in the microsomal membrane fraction, whereas only a band of smaller molecular weight is recognized by the same antibody in the endomembranes fraction. This is a strong indication that NtSUT4 occurs in two different forms depending on its subcellular localization.

### StSUT4-GFP is not exclusively localized to the plasma membrane of stably transformed potato plants

**Figure 2:**
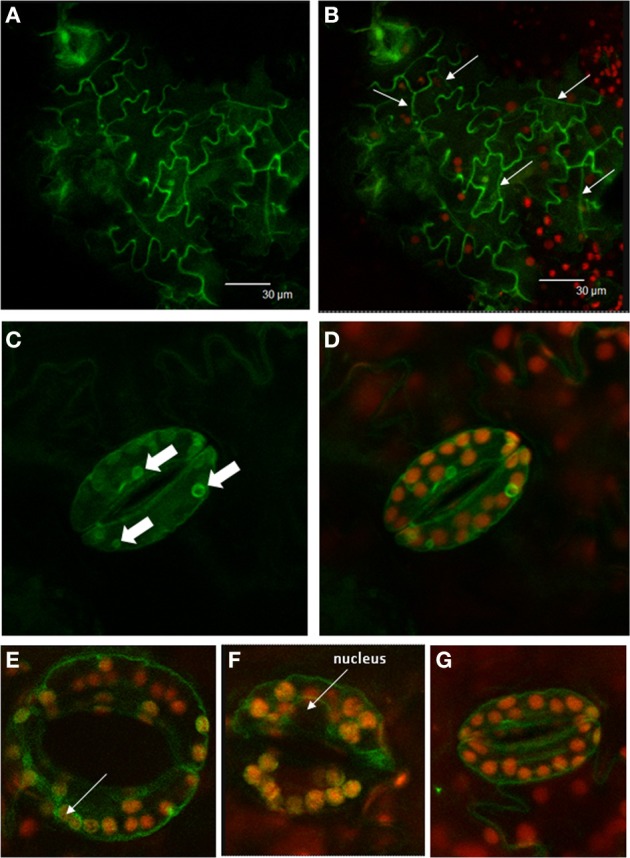
**Localization of StSUT4-GFP in stably transformed potato plants.** *Solanum tuberosum* andigena has been transformed with StSUT4-GFP in pCK205 used previously for transient transformation (Chincinska et al., 2008). **(A)** StSUT4-GFP in young developing sink leaves. **(B)** Same picture as shown in **(A)** overlaid with chlorophyll autofluorescence. Arrows mark newly formed cell walls. Epidermis cells undergoing actively cell division. **(C)** StSUT4-GFP expression in mature source leaves is mainly restricted to guard cells. Arrows mark intracellular fluorescently labeled structures. **(D)** Same area as shown in **(C)** overlaid with chlorophyll autofluorescence indicating plastid localization. Note that GFP fluorescence is detectable outside of chloroplasts. **(E–G)** Guard cells of source leaves showing StSUT4-GFP fluorescence in the periphery of the cells, but sparing the region where the nucleus is localized (arrow) arguing for vacuolar localization.

*StSUT4-GFP* fusion constructs have been used previously for transient expression in tobacco and potato leaves by infiltration and subsequent subcellular localization (Chincinska et al., 2008). StSUT4-GFP was localized to the plasma membrane, and to membranes surrounding the nucleus, most likely the ER. The *StSUT4* mRNA stability is regulated at the post-transcriptional level and is obviously stabilized under far-red light enrichment (He et al., 2008; Liesche et al., 2011). Although expressed under the constitutive 35S-promoter it is interesting to study *StSUT4-GFP* expression in stably transformed plants in order to analyse its post-transcriptional regulation, especially during plant development. *Solanum tuberosum* ssp. *andigena* plants have been stably transformed with the same StSUT4 construct used before for transient transformation (Chincinska et al., 2008). Interestingly, StSUT4 is detectable at the plasma membrane mainly in sink leaves of young potato plantlets (Figures 2A,B). The epidermal cells expressing the *StSUT4-GFP* fusion construct seem to be actively undergoing cell division. Later during plant development, *StSUT4-GFP* expression is hardly detectable and mainly confined to the guard cells. Here, the labeling is no longer detectable at the plasma membrane. The fact that the GFP labeling in the cell periphery spares the nuclear region (Figures 2F,G) or the region of peripheral chloroplasts (Figure 2E) is a good argument for vacuolar localization. Smaller vacuolar compartments with the size of chloroplasts also reveal GFP fluorescence in mature source leaves of stably transformed potato plants (Figures 2C,D).

### SUT4 reduces sucrose sensitivity

**Figure 3:**
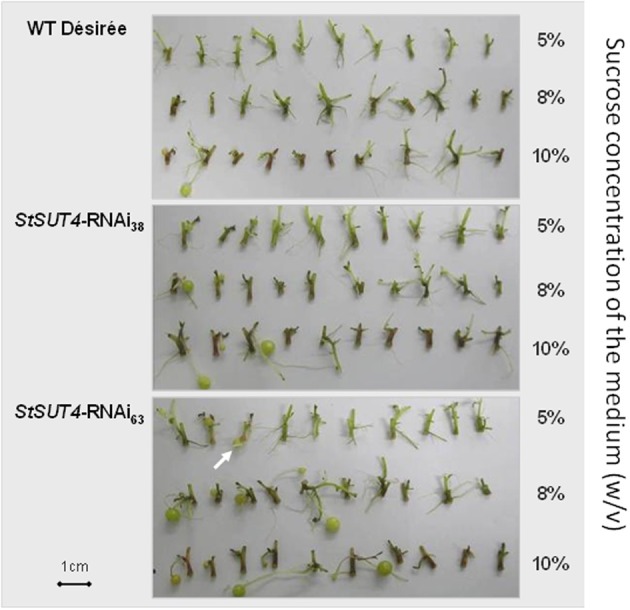
**Microtuber production in response to various sucrose concentrations in the dark.** Stem cuttings from two independent transgenic lines (StSUT4-RNAi38 and StSUT4-RNAi 63) and WT potato plants from sterile tissue culture were transferred in MS-medium containing 5, 8, or 10% sucrose (w/v). Pictures were taken 10 days after transfer into the dark. The experiment was repeated three times independent experiments and one representative example is given. Note that microtuber production in the transgenic line StSUt4-RNAi #63 is induced by 5% sucrose (arrow). Microtuber induction experiments were performed with *Solanum tuberosum* ssp. tuberosum plants of the variety Désirée.

The StSUT4-inhibited plants also show an increased carbon export from source leaves at the end of the light period when sucrose export arrives to its maximum (Chincinska et al., 2008). This increase in sucrose efflux correlates with significantly elevated sucrose and starch levels in sink organs such as *in vitro* grown microtubers. Here, the influence of exogenously supplied sucrose on tuberization in *StSUT4* RNAi and wild type plants was tested by an *in vitro* tuberization assay. When grown in darkness, which excludes leaf carbon export effects, the induction of microtubers depended on the concentration of sucrose in the medium. Interestingly, tuber induction in *StSUT4*-inhibited plants was induced even in the presence of 5% or 8% of exogenous sucrose, whereas wild type potato stem cuttings start tuberization only at 10% of sucrose concentration (Figure 3). Thus, not only the sucrose supply to the terminal sink organs seems to be affected by StSUT4, but also the sucrose sensitivity toward the substrate sucrose. StSUT4 seems to decrease sucrose sensitivity and sucrose efflux in wild type plants under normal growth conditions.

### SUT4 affects ethylene production

The impact of ethylene on flowering, tuberization, and shade avoidance response has been investigated further by some more recent studies (Wuriyanghan et al., 2009; Stamm and Kumar, 2010). Key enzymes of ethylene and gibberellin biosynthesis, the ACC oxidase and the GA20oxidase1 have therefore been analysed in more details and transcript levels of both key enzymes were shown to be significantly reduced in *StSUT4*-inhibited plants (Chincinska et al., 2008). Determination of the production of the gaseous phytohormones ethylene was performed within the Trace Gas Facility of the University of Nijmegen using a portable Ethylene Sensor Sense. This device is equipped with a laser-based ethylene detector connected to a gas flow through system using air tight cuvettes. The high sensitivity of the system allows to measure ethylene amounts in the nanoliter range of a rather weak ethylene producer like potato plants. Compared to tomato plants, potato is a crop producing only very low amounts of ethylene in the range of 0.001–0.1 μl/kg fresh weight × h (Alders, 1987; Wheeler et al., 2004).

**Figure 4:**
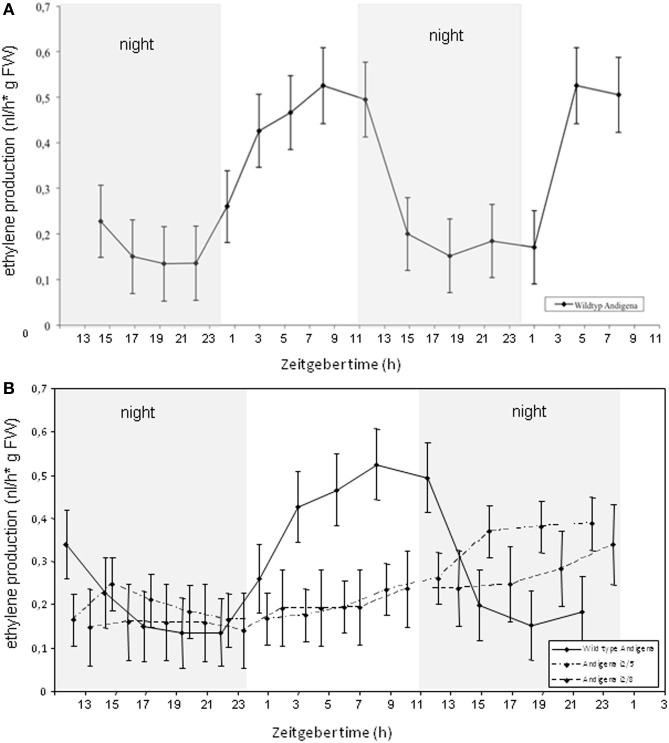
**(A)** Ethylene production in *Solanum tuberosum* ssp. andigena plants over a 2 days period show diurnal rhythm with increasing ethylene production during day time and decreasing ethylene production during the night. Plants were grown under day neutral conditions (12 h light/12 h darkness, grey bars indicate dark periods). **(B)** Ethylene production in StSUT4-RNAi plants # i2/5 and #i2/8 is significantly lower than in potato wild type plants and does not follow diurnal oscillation pattern. Experiments were repeated twice and a representative example is given. The ethylene production is given in nanoliters per hour per gram fresh weight. Error bars indicate the StDev.

**Figure A1:**
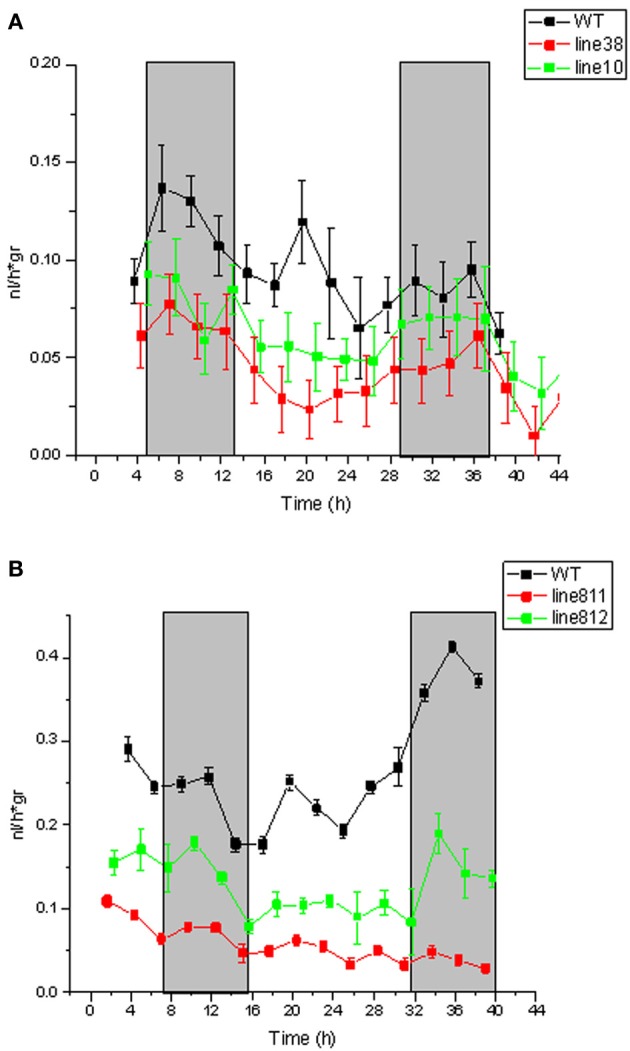
**Ethylene production in wild type Désirée and StSUT4-RNAi potato plants.** Tissue cultured plants of wild type *Solanum tuberosum tuberosum* Désirée and corresponding StSUT4-RNAi lines #38, #10 (in **A**) and two different plants of the transgenic line StSUT4-RNAi #81 (in **B**) have been analyzed. Time is in h and the grey areas indicate the dark period during the experiment. Plant were grown under long day conditions (16 h light, white light of 270 μmol/m^2^ × s, grey bars indicate dark periods). Ethylene production is given in nl per h per g fresh weight. Tested plants were kept 3–4 weeks in 2MS medium before ethylene measurement. Fresh weight of plants: A, WT 2.49 g, StSUT4-RNAi #38 1.86 g, StSUT4-RNAi #10 1.84 g. B, WT 3.86 g, StSUT4-RNAi #81.1 5.44 g, StSUT4-RNAi #81.2 4.17 g. StDev is given.

*StSUT4*-inhibited potato plants of the subspecies *tuberosum* as well as of the strict photoperiodic subspecies *andigena* have been analysed using the ethylene detection system (Figures 4, A1). Whereas *andigena* WT plants show a diurnal rhythm of ethylene production, the ethylene production of *S. tuberosum* Désirée WT plants did not follow a diurnal pattern (Figure A1). StSUT4-inhibited *S. tuberosum* andigena plants do not show rhythmic ethylene production as shown for wild-type control plants (Figure 4) and the total amount of ethylene production of *StSUT4*-inhibited plants seems to be reduced compared to the wild-type potato plants in both subspecies (Figures 4, A1). Reduced ethylene production might be the reason for the lack of shade avoidance response in these plants (Chincinska et al., 2008).

### SUT4 affects the expression of flowering-related genes in a photoperiod-dependent manner

Previous studies revealed an early flowering and tuberization of *StSUT4*-inhibited potato plants in both andigena and tuberosum subspecies (Chincinska et al., 2008). Flowering and tuberization in potato share common pathways and genes (Rodriguez-Falcon et al., 2006; Kühn, 2011). The circadian-regulated homologous genes such as *StCONSTANS*, *flowering locus T*, *Suppressor of CONSTANS* (*SOC1*) and *GIGANTEA* were concluded to be involved in the photoperiod-dependent induction of tuberization in potato (Rutitzky et al., 2009; Navarro et al., 2011; Gonzalez-Schain et al., 2012). Consequently, it was proposed that the flower and tuber induction are highly conserved pathways which use identical molecules (Martinez-Garcia et al., 2002).

The phenotypical differences of *StSUT4*-inhibited potato plants compared to WT plants is restricted to non-inductive long day conditions, whereas under short day conditions, when WT potato plants are induced to produce tubers, the inhibition of *StSUT4* expression seems to be less important.

**Figure 5:**
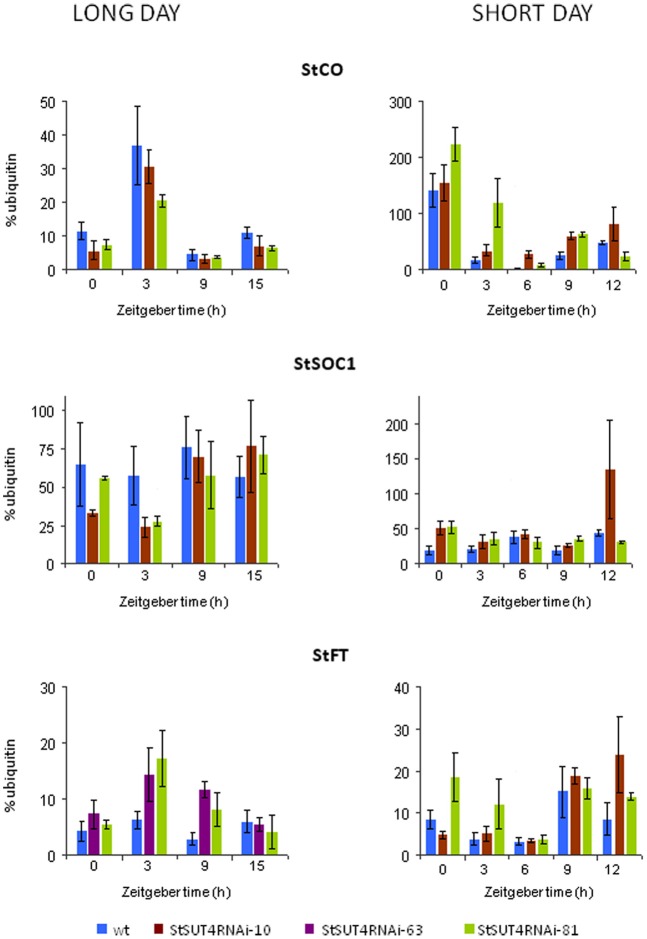
**Quantification of the transcript levels of circadian-regulated gene *StFT, StSOC1*, and *StCO* via reverse transcription real time PCR in leaves of *Solanum tuberosum* Désirée plants.** Potato plants were grown either under short day (10 h light with a light period from 8 a.m. to 6 p.m.) or long day (16 h light with a light period from 6 a.m. to 10 p.m.) conditions. *StCO* and *StSOC1* mRNA accumulation is reduced in *StSUT4*-inhibited plants under short day conditions, but increased under long day conditions. StFT transcript levels are increased under both cultivation conditions, short day as well as long day conditions. Relative expression levels are given using ubiquitin mRNA as internal standard. Two technical replicates are performed from two biological replicates in each case. Experiments were repeated at least twice. One representative example is given.

**Figure 6:**
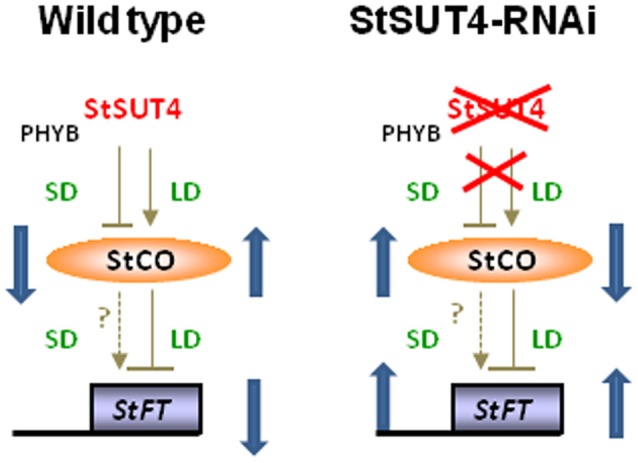
**Hypothetical model illustrating the assumed impact of the circadian-regulated gene StSUT4 on the photoperiod-dependent accumulation of StCONSTANS (StCO) and flowering locus T (StFT) transcript levels.** Whereas in WT plants StSUT4 inhibits StFT accumulation under long day conditions, this photoperiod-dependent regulation via the StCONSTANS protein is deregulated in *StSUT4*-inhibited plants leading to increased StFT levels under both, long day and short day conditions leading to early flowering and tuberization even under non-inductive conditions.

In order to test the hypothesis that *StSUT4* expression affects flowering and tuberization induction photoperiodically, the transcript levels of known circadian-regulated genes from potato, namely *StCO, StFT* (or *StSP6*), and *StSOC1*, have been quantified by reverse transcription real time PCR. As shown in Figure 5 consistent differences were found for the transcript levels of these genes between wild-type and StSUT4 down regulated plants (Figure 5). The *StCO* transcript levels of *StSUT4*-inhibited plants decreased under long day conditions when compared to the wild-type plants, while the increase was observed under the short day conditions. This tendency is paralleled by the level of *StSOC1*. Under long day conditions, high levels of *StCO* mRNA during the light period (thereby enabling StCO protein synthesis) have inhibitory effect on the level of *StFT* mRNA in short day plants. Therefore, short day-dependent tuberization in potato plants (as well as short-day dependent flowering in SD plants) is normally inhibited under long day conditions. Since in *StSUT4*-inhibited plants, the level of *StCO* mRNA is decreased under LD conditions, the inhibitory effect on *StFT* mRNA levels is diminished, leading to abnormal levels of *StFT* mRNA under non-inductive conditions. This deregulation of flowering and tuber inducing gene expression is assumed to be responsible for tuberization of *StSUT4*-inhibited plants even under non-inductive long day conditions. SUT4 could therefore be a potential link between expression of *StCO, StFT*, and *StSOC1* and day length. All phenomena are summarized in a hypothetical model shown in Figure 6.

## Discussion

### Heterogeneity of the SUT4 transporter localization

Although the function of the SUT4 sucrose transporters seem to be highly diverse, many members of this phylogenetic clade seem to undergo dual targeting to the tonoplast and the plasma membrane (Table 1).

Recently, several putative tonoplast sorting signals have been described in plants, which have similarities to animal or yeast targeting motifs. Tonoplast and lysosome targeting signals include tyrosine-based YXX∅ motifs (with ∅ representing a bulky amino acid residue) and acidic dileucine motifs (D/E) XXX L (L/I) in the C-terminal or N-terminal domain. An acidic dileucine motif in the C- or N-terminal domain seems to be necessary and sufficient for tonoplast targeting of peptide, glucose, or inositol transporters (Komarova et al., 2012). However, sorting signals such as tyrosine and dileucine based motifs can also be present in plasma membrane proteins where they are involved in the internalization by endocytosis (Irani and Russinova, 2009).

AtSUC4 from Arabidopsis, which was found in the vacuole, does not contain any of the putative sorting signals known from animals or other plants (Wolfenstetter et al., 2012). AtSUC4 follows a non-classical sorting pathway which depends on the AP-3 adaptor protein complex and which is potentially involved in the sorting to lytic vacuoles. A plant-specific role for the evolutionarily conserved AP-3 adaptor complex in mediating lytic vacuole performance and transition of storage into the lytic vacuoles is suggested (Feraru et al., 2010).

Western blot analysis of potato leaves following SUT4 immunodetection suggests the presence of a truncated version of the SUT4 protein in the endomembrane fraction, whereas the full length protein is exclusively detectable in the plasma membrane fraction (Chincinska et al., 2008). It should be noted that members of the SUT4 family of sucrose transporters are degraded in lytic vacuoles to guarantee a rapid and tightly controlled turnover.

The function of StSUT4 localized at the internal membranes, as seen in guard cells of mature leaves (Figures 2C–G), remains to be elucidated. The localization at the internal membranes and small vacuoles does not resemble the targeting of SUT4 to the tonoplast, which has been reported for other plant species. Turn-over of the transporter would have resulted in the complete loss of signal, as seen in the other epidermal cells of the leaf. It is possible that SUT4 is involved in the regulation of a subcellular sucrose distribution, which could be important for the osmotic pressure regulation in guard cells. However, truncation of the SUT4 protein in the endomembrane fraction suggests that the SUT4 protein undergoes degradation in lytic vacuoles. To verify the localization in vacuole-like compartments in guard cells, SUT4-GFP lines using the native promoter should be examined in order to exclude overexpression effects. Ideally, the use of an antibody that is specific for the full protein should explain where the functional SUT4 can be found within the cell.

### Potential sink-specific function of StSUT4 in sucrose sensing

The physiological consequences of down-regulated SUT4 expression are diverse. The StSUT4 knock-down has different effects than inhibition or knock-out of OsSUT2, PtaSUT4, or AtSUC4. This effect could be due to the different functions of SUT4 in plants with different phloem loading strategies. Whereas poplar and rice load sucrose symplasmically, i.e., sucrose diffuses through plasmodesmata into the sieve element-companion cell complex, according to the cytosolic concentration potential (Fu et al., 2011; Eom et al., 2012), potato is an apoplastic loader, in which the phloem is symplasmically isolated and sucrose has to be taken up from the apoplast via membrane transporters.

Here, we report possibly a new function of StSUT4 in sink organs. The *in vitro* experiments (Figure 3) revealed altered sucrose-sensitivity of potato stolons, which could be indicative of a StSUT4 function in sucrose sensing. However, this effect could be also due to the increased StSUT1 expression, because StSUT1 was suggested to be negatively regulated by StSUT4 (Chincinska et al., 2008). Increased StSUT1 levels could increase the uptake of sucrose into the stolon, allowing for tuberization at lower sucrose levels.

### Sucrose transport, ethylene signaling, and flowering

Involvement of sucrose transporters in ethylene signaling was already described in other plant species. Recently it was shown that the regulation of sucrose transporter AtSUC1 expression by ethylene inhibits sucrose-induced anthocyanin accumulation in the presence of light (Jeong et al., 2010). Anthocyanin accumulation is positively affected by sugar and light and negatively affected by ethylene. The expression of AtSUC1 is enhanced in ethylene insensitive mutants of Arabidopsis (*etr1-1, ein2-1, ein3-like1*) and by treatment with silver ions, which are known to inhibit ethylene perception by the ethylene receptors. AtSUC1 is assumed to play a role as an integrator for signals transmitted by sugar, light, and ethylene (Jeong et al., 2010).

In Arabidopsis, the CONSTANS protein responsible for promoting flowering, affects early targets such as SOC1 and FT, but also other target genes involved in proline or ethylene biosynthesis (Samach et al., 2000). Ethylene was shown to delay flowering, possibly by modulating DELLA activity (Achard et al., 2007). Ethylene-enhanced DELLA accumulation was assumed to delay flowering via repression of LEAFY (LFY) and SUPPRESSOR OF OVEREXPRESSION OF CONSTANS 1 (SOC1) (Achard et al., 2007). The same effect was observed in rice plants, where the overexpression of the ethylene receptor ETR2 reduced ethylene sensitivity and delayed floral transition (Wuriyanghan et al., 2009).

The role of sugars in flowering is widely accepted. In this context members of the INDETERMINATE DOMAIN (IDD) transcription factors that are involved in floral transition were described to modulate expression of sucrose transporter and sucrose metabolizing enzymes. The expression of genes encoding sucrose transporters AtSUC2, AtSUC6, AtSUC7, and AtSUC8, as well as of genes encoding sucrose synthases and invertases are affected by the expression of the IDD transcription factor IDD8 in Arabidopsis (Seo et al., 2011). *IDD8* expression also affects the expression of the flowering genes *FT* and *SOC1* and corresponding *idd8* knock out mutants show a late flowering phenotype. The IDD8 transcription factor binds directly to the SUS4 promoter and responds to photoperiodic signals. Sugar transport and metabolism is therefore tightly linked to the photoperiodic flowering.

In potato plants, StCONSTANS protein is involved in the photoperiodic control of the flowering as well as for the tuberization. StCO represses tuberization in a photoperiod-depended manner and affects the phloem-mobile *StBEL5* mRNA, which promotes tuberization. Thus, StCO regulated long-distance signaling molecules in potato as well (Gonzalez-Schain et al., 2012). We suggest that StSUT4 affects the accumulation of *StCO* and *StFT* mRNA in a photoperiod-dependent manner, as it is illustrated in Figure 6. Due to the lack of StSUT4, the accumulation of *StCO* mRNA under SD conditions is not inhibited, leading to increased levels of *StCO* mRNA in *StSUT4*-inhibited plants. Since the inhibitory effect of StCO on *StFT* mRNA accumulation is assumed to occur only under LD but not under SD conditions, this increase is not accompanied by a decrease of *StFT*, leading to increased levels of StFT mRNA under both growth conditions.

Recently, a detailed macromolecular analysis revealed the presence of ethylene biosynthetic components in the phloem sap of *Lupinus albus* (Atkins et al., 2011). Since all of the known sucrose transporters from potato are phloem-localized, it can be assumed that sucrose transporter expression affects ethylene production in the phloem.

StSUT4 follows a circadian expression pattern, affects flowering, tuberization, and shade avoidance (Chincinska et al., 2008). By determination of a reduced rate of ethylene biosynthesis of *StSUT4*-inhibited plants, we provide first evidence for a potential link between photoperiodic flowering control and ethylene biosynthesis via sucrose transporter like protein, StSUT4.

Presently, it cannot be excluded that the effect of ethylene synthesis on the flowering behavior of potato plants involves the gibberellin-dependent pathway, since key enzymes of the gibberellin biosynthesis are also down-regulated (Chincinska et al., 2008). The important role and interplay between gibberellins and sucrose in the floral initiation has been described (Blazquez et al., 1998; King and Ben-Tal, 2001; Eriksson et al., 2006).

## Methods

### Plant growth conditions and tissue culture

Potato plants in sterile culture were grown on 2MS-medium (MS-medium according to Murashige and Skoog, 1962 with 2% sucrose) in tissue culture chambers at 24°C, at 50% humidity and 1000 μmol photons m^−2^ s^−1^ with a light/dark cycle of 16 h/8 h (long day) or 10 h/12 h (short day).

### Recombinant DNA

Generation of the StSUT4-GFP construct was described earlier (Chincinska et al., 2008). Cloning of the StSUT4 cDNA into the yeast expression vector pDR196 GW kindly provided by Doris Rentsch (Bern, Switzerland) was done using the GATEWAY technology (Invitrogen) by help of the primers: StSUT4 attB1 fw: AA AAA GCA GGC TTA ATG CCG GAG ATA GAA AGG CAT AG, StSUT4 attB2 rev: A GAA AGC TGG GTT TCA TGC AAA GAT CTT GGG TTT C.

### Plant transformation

Stable transformation of *Solanum tuberosum* Désirée was performed with *Agrobacterium tumefaciens* [strain C58C1, pGV2260; (Deblaere et al., 1985)] with small modifications according to Rocha-Sosa et al. (1989). Transformation was confirmed by PCR analysis and test for GFP fluorescence by CLSM (Leica, TCP SP2).

### Western-blot analysis

Isolation of the microsomal fraction from plant material as well as two-phase partitioning and western blotting were performed as previously described (Lemoine et al., 1996). The StSUT4-specific peptide antibody is raised against a central loop peptide of SUT4 (NH_2_-CGSSHTGEEIDESSHGQEEAFLW-CONH_2_). The specificity of the affinity-purified antibody has been tested here and elsewhere (Weise et al., 2000).

### *In vitro* tuberization assay

Stem segments including at least one node of 6 weeks old sterile potato plants were prepared under sterile conditions and planted on MS medium containing 10% sucrose. After 1 week under LD conditions in the growth chamber (16 h light, 8 h darkness, 24°C), the scions were transferred into darkness to induce tubers. *In vitro* tubers were harvested after 20 days.

### Greenhouse

Transgenic plants were amplified in tissue culture, transferred to soil and grown in a cycle of 16 h light (22°C) and 8 h darkness (15°C) in 60% humidity. The mean photosynthetic photon flux density (PPFD; 400–700 nm) was about 150 μmol photons m^−2^ s^−1^ and additional illumination was provided by high-pressure sodium lamps SON-T Green Power and metal halide lamps MASTER LPI-T Plus (Philips Belgium, Brussels). Emitted light from Philips SON-T Green Power has a red: far-red ratio (660/730 nm) of 2.63 and from Philips HPI-T Plus of 1.25. Both lamps are distributed equally in the green house.

### RNA quantification by reverse transcription real-time PCR

RNA was isolated from different organs of greenhouse grown *S. tuberosum* Désirée and andigena or from leaf discs of potato plants grown in the phytochamber. RNA extraction was performed with Trisure (Bioline, Luckenwalde, Germany) or peqGold Trifast (Peqlab, Erlangen, Germany) according to the manufacturer's protocol. Reverse transcription was performed with the Qiagen Omniscript RT Kit according to the manual. Optimized conditions included using oligo(dT) primers for the initial reverse transcription reaction on approximately 1 μg of total RNA after digestion with RNase-free DNase (Qiagen, Hilden, Germany).

Aliquots of 0.2 μl of the 10 μl RT-reaction were used for the subsequent PCR reaction in the presence of SYBR Green with HotGoldStar DNA Polymerase (Eurogentec, Seraing, Belgium) in a Rotor Gene 3000 Cycler (LTF Labortechnik, Wasserburg, Germany) using the Rotor Gene Software Version 4.6.94. The best products were obtained with the following program: denaturation at 95°C for 30 s, annealing for 30 s at 61°C and elongation for 30 s at 72°C, in a program of 45 cycles in 20 μl reaction volume. Relative quantification of transcript amounts was always calculated in relation to the respective *ubiquitin* transcript level and given in % of *ubiquitin*. Primers were designed to obtain a 50–150 bp amplicon using Primer3 software (http://frodo.wi.mit.edu/cgi-bin/primer3/primer3_www.cgi).

Primer sequences used for reverse transcription real time PCR analysis: *Ubiquitin* fw: CAC CAA GCC AAA GAA GAT CA, *Ubiquitin* rev: TCA GCA TTA GGG CAC TCC TT; *LC-SUT1* fw: TTC CAT AGC TGC TGG TGT TC; *LC-SUT1* rev: TAC CAG AAA TGG GTC CAC AA; *StSUT4* fw: GCT CTT GGG CTT GGA CAA GGC; *StSUT4* rev: GGC TGG TGA ATT GCC TCC ACC; *StFT* fw: GTG GAT CCT GAT GCT CCA AG; *StFT* rev: TTC CTG TGG TTG CTG GGA TA; *StCO* fw: CTT CAA ACT CCC ATC CAC GA; *StCO* rev: TTG GAG TAA GCT GGG GAG GT; *StSOC1* fw: TCC AGC ACG CAG GAG ATA AT; *StSOC1* rev: CCA GCT TGG TTT TCA GGT TG; *StGI* fw: GCT TCC TCC ACA AGA TG; *StGI* rev: TGG ATA CCG GTT CCG TAT GA. Reverse transcription real time PCR data were corrected by calculation of the PCR efficiency individually using the LinReg PCR software.

### Real-time monitoring of ethylene production

Plants were measured over a period of up to 2 days in 6 air tight glass cuvettes on MS medium. Empty glass cuvettes with MS medium only were subtracted from the measurements. Measurements were done in a chamber with constant temperature (Sanyo MLR-350H; Light source: Phillips, TL-D-36W/33-640SLV). The chamber was adjusted to a light dark cycle with 11 h light/13 h darkness and a temperature of 21°C.

Ethylene production was measured with a laser-based ethylene detector (ETD-300; Sensor Sense BV; Nijmegen, Netherlands). A detailed description of the system has been given elsewhere (Cristescu et al., 2008). Briefly, the detector consists of a CO_2_ laser emitting radiation in the 10-μm infrared wavelength region and a photoacoustic cell, in which ethylene is detected. The detector makes use of the distinct fingerprint-like absorption features of ethylene in the CO_2_ laser wavelength range (Cristescu et al., 2002). Inside the photoacoustic cell traces of ethylene can absorb the laser radiation; the absorbed energy is released into heat, which creates an increase in pressure inside a closed volume. By modulating the laser beam, pressure waves (i.e., sound) are generated and detected with a sensitive miniature microphone. The amplitude of the acoustic waves is directly proportional to the concentration of ethylene in the photoacoustic cell. A gas flow through system (VC-6, Sensor Sense BV; Nijmegen, Netherlands) allowed automated sampling of ethylene production under a stop-and-flow mode. Ethylene production of 6 cuvettes was accumulated during 3 h and then was flushed into the detector for 30 min at a flow rate of 3 l/h. A scrubber with KOH (moist pellets) was used to reduce the CO_2_ concentration to less than 1 ppm, and a tube with CaCl_2_ (granules) was placed directly after this scrubber in order to decrease the water content in the gas flow. The ethylene production by the potato plants was expressed in nanoliters per hour per gram (fresh weight). Each experiment was repeated twice, and representative data are shown below.

### Conflict of interest statement

The authors declare that the research was conducted in the absence of any commercial or financial relationships that could be construed as a potential conflict of interest.
